# Sex Differences in Frequency of Instrumental Activities of Daily Living after Cardiac Rehabilitation and Its Impact on Outcomes in Patients with Heart Failure

**DOI:** 10.3390/jcdd9090289

**Published:** 2022-08-31

**Authors:** Kohei Nozaki, Nobuaki Hamazaki, Kentaro Kamiya, Hidenori Kariya, Shota Uchida, Takumi Noda, Kensuke Ueno, Emi Maekawa, Atsuhiko Matsunaga, Minako Yamaoka-Tojo, Junya Ako

**Affiliations:** 1 Department of Rehabilitation, Kitasato University Hospital, 1-15-1 Kitasato, Minami-ku, Sagamihara 252-0375, Japan; 2 Department of Rehabilitation, School of Allied Health Sciences, Kitasato University, Sagamihara 252-0373, Japan; 3 Department of Rehabilitation Sciences, Kitasato University Graduate School of Medical Sciences, Sagamihara 252-0373, Japan; 4 Department of Cardiovascular Medicine, Kitasato University School of Medicine, Sagamihara 252-0374, Japan

**Keywords:** instrumental activities of daily living, sex difference, heart failure, outcome, cardiac rehabilitation

## Abstract

Although instrumental activities of daily living (IADL) are included in the outcomes of cardiac rehabilitation (CR), the relationship between IADL frequency at the end of CR and outcomes between the sexes remains unclear. We aimed to investigate the differences in frequency of IADL between the sexes and its impact on the outcomes. We retrospectively investigated 490 consecutive patients who were admitted for heart failure (HF) and participated in CR post-discharge. IADL frequency was assessed using the questionnaire-based Frenchay Activities Index (FAI). The primary endpoint was all-cause death, and the secondary endpoint was a composite of all-cause death and readmission due to HF. The cut-off values of the FAI for all-cause death in the overall cohort, females, and males were 23, 22, and 23 points, respectively. After adjusting for several factors, IADL assessed using the FAI was independently associated with all-cause mortality (hazard ratio [HR]: 0.961, 95% confidence interval [CI]: 0.937–0.986) and combined events (HR: 0.968, 95% CI: 0.952–0.985), respectively. Additionally, there was no interaction between sex and all-cause mortality. In conclusion, higher IADL frequency after CR was associated with favourable outcomes in patients with HF.

## 1. Introduction

The number of patients suffering from heart failure (HF) is increasing worldwide, and it is a serious public health problem [1,2]. In these patients, treatment modalities for HF are advancing, and cardiac rehabilitation is known to improve their outcomes [3,4].

While the major outcomes of cardiac rehabilitation include improvements in exercise tolerance, prognosis, and quality of life [5], instrumental activities of daily living (IADL) are also among the outcomes [6]. IADL include doing laundry, shopping, going out, and other activities that are important for independent daily living [7]. Although IADL require a higher level of physical performance than basic activities of daily living, most patients are expected to continue them for a long time [8]. IADL have been reported to be associated with all-cause mortality and quality of life in community-dwelling people and in patients with stable heart disease [9,10,11,12]. However, no report has examined the relationship between IADL after cardiac rehabilitation for HF and the outcomes. Additionally, it is assumed that the differences between the sexes may affect the frequency of performing each component of IADL; however, there are no reports that have examined its association with outcomes between the sexes. To better comprehend the importance of a high level of IADL as an outcome of cardiac rehabilitation, it is necessary to clarify the relationship between IADL and the outcomes at the end of cardiac rehabilitation while incorporating the differences between the sexes.

Therefore, the purpose of this study was to investigate the relationship between IADL at the end of cardiac rehabilitation and the outcomes in patients who were hospitalized for HF and participated in outpatient rehabilitation post-discharge separately between the sexes.

## 2. Materials and Methods

### 2.1. Study Population

A retrospective review was performed in an initial population of 717 consecutive patients admitted to our hospital due to acute decompensated HF between January 2010 and May 2015 who had participated in outpatient cardiac rehabilitation. The exclusion criteria were as follows: (1) missing IADL assessments; (2) morbidities that necessitate assistance in daily life; (3) hemodialysis; and (4) readmission or death before the end of outpatient cardiac rehabilitation. Acute decompensated HF was defined according to the Framingham criteria [13]. Patients who participated in the outpatient rehabilitation underwent a programme consisting of 20–40 min of exercise therapy and disease management education once a week to once a month for approximately 3 months after the hospital discharge under the supervision of a doctor, physical therapist, and nurse using a standardised protocol designed according to the Japanese Circulation Society guidelines for the treatment of HF [14]. This study was performed in accordance with the tenets of the Declaration of Helsinki, and the protocol was approved by the ethics committee of our institution (KMEO B18-075). Verbal informed consent was obtained from all participants, and the information regarding the study was made public using the opt-out method [15].

### 2.2. Data Collection

Data were collected from the patients’ electronic medical records. Clinical data including biochemical and echocardiographic data were measured at the end of outpatient cardiac rehabilitation. For measuring the serum concentration of B-type natriuretic peptide (BNP), blood samples were promptly stored in ice-cold storage and calculated in a laboratory in our hospital using a commercially available immunoradiometric assay (Shionogi, Osaka, Japan). The estimated glomerular filtration rate (eGFR) was calculated using the formula recommended by the Japanese Society of Nephrology: 194 × (serum creatinine)^1.094^ × (age)^0.287^ in men and 194 × (serum creatinine)^1.094^ × (age)^0.287^ × 0.739 in women [16]. The left ventricular ejection fraction (LVEF) was estimated using Simpson’s method on two-dimensional echocardiograms.

### 2.3. IADL Measurement

IADL frequency was assessed using the Frenchay Activities Index (FAI) at one point at the end of the outpatient cardiac rehabilitation, which was taken as the baseline [17]. The FAI contains 15 items, including preparation of main meals, washing up, washing clothes, light housework, heavy housework, local shopping, social outings, walking outside for >15 min, actively pursuing hobbies, driving a car or bus travel, outings or car rides, gardening, household or car maintenance, reading books, and gainful work. Each item is scored on a four-point scale (0–3), and the score ranges from 0 to 45 points. A higher score indicates a higher IADL.

### 2.4. Outcomes

The primary outcome of this study was all-cause death, and the secondary outcome was a composite endpoint of rehospitalization due to HF and all-cause death. The time for the endpoint was calculated as the number of days from the end of the outpatient cardiac rehabilitation to the date of the event.

### 2.5. Statistical Analyses

The results of normally distributed continuous variables are expressed as median (interquartile range [IQR]). Categorical variables are expressed as numbers and percentages. The distributions of the FAI between the sexes were examined, and the sub-items of the FAI were compared between them. We constructed the receiver-operating characteristic (ROC) curves of the FAI for all-cause mortality according to sex, and the area under the curve (AUC) was calculated. According to the AUCs, we calculated the Youden index to determine the cut-off points of the FAI for all-cause mortality in females and males. The patients were divided into the low FAI group and the high FAI group according to the cut-off points. The baseline characteristics were compared between the groups using the Mann–Whitney U test for continuous variables and the Chi-square test for categorical variables.

The effects of FAI on survival were examined using the Kaplan–Meier method using the log-rank test in the overall cohort as well as in males and females separately. Additionally, multivariable Cox regression analyses were performed for the FAI adjusted for the age, sex, body mass index (BMI), New York Heart Association (NYHA) functional classification Ⅲ/Ⅳ, LVEF, prior HF readmission, log-transferred BNP, β-blockers, and the AHEAD score. The AHEAD sore includes atrial fibrillation, hemoglobin, age, creatinine, and the presence of diabetes mellitus. Its discrimination and calibration have been well validated in Asian patients with HF. Additionally, histograms of the FAI total scores were plotted for females and males to evaluate the distribution of the frequency of IADLs performed. The frequency of performing each of the FAI components was also compared between females and males using the χ-square test.

Furthermore, multivariate Cox proportional regression analysis adjusted for the propensity score was performed as sensitivity analysis because the number of adjustment variables used was large for the number of events as well as to adjust for overfitting. The propensity score was calculated using multivariate logistic regression analysis based on the age, sex, BMI, NYHA functional classification Ⅲ/Ⅳ, LVEF, prior HF readmission, log-transferred BNP, β-blockers, and the AHEAD score. Furthermore, to investigate the potential effect modification on the association of the pupil area with mortality, we performed subgroup analyses of the pupil area in various subgroups relevant to HF prognosis, including the sex, age (stratified at 65 and 75 years), LVEF (stratified at 40% and 50%), and history of prior HF admission with adjustments for the same confounding factors.

Multiple imputation was used to take into account the missing covariate data to construct multivariable Cox regression models. We created 20 datasets using a chained-equations procedure. Parameter estimates were obtained for each dataset and subsequently combined to produce an integrated result using the method described by Barnard and Rubin [18].

Analyses were performed using SPSS version 25.0 (IBM Corporation, Armonk, NY, USA), Stata version 15.0 (StataCorp LP, College Station, TX, USA), and R version 2.5-1 (R Foundation for Statistical Computing, Vienna, Austria). In all analyses, a two-tailed *p* < 0.05 was taken to indicate statistical significance.

## 3. Results

### 3.1. Study Population

After excluding the patients with missing FAI assessments (*n* = 130), those that required assistance in daily life (*n* = 52), those on hemodialysis (*n* = 4), and those with readmission or death before the end of cardiac rehabilitation (*n* = 41), 490 patients were included in the analysis.

Table 1 summarises the baseline characteristics of all patients. The median age was 69 years, and 33.9% of the patients were females. The median value of LVEF, serum BNP, and AHEAD score was 48%, 219 pg/dL, and 2 points, respectively. Additionally, 85% of all patients were prescribed angiotensin-converting enzyme inhibitors (ACEIs) or angiotensin II receptor blockers (ARBs), 77% were prescribed β-blockers, and 45% received mineralocorticoid receptor antagonists (MRAs). In this study population, the median FAI score was 24 points.

### 3.2. Differences in the Distribution of Total FAI Scores and Each of the Component Items between the Sexes

**Figure 1:**
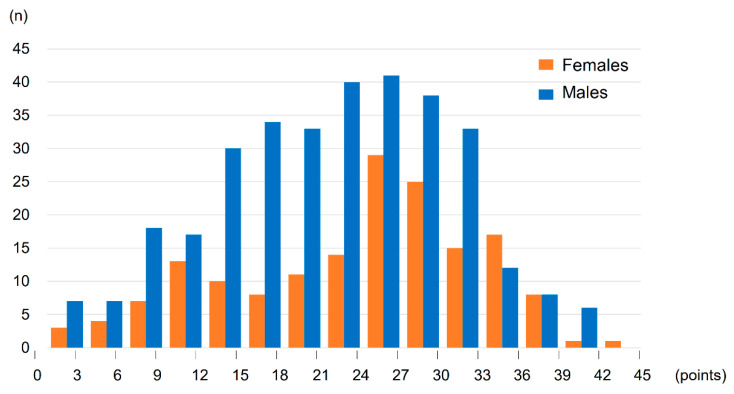
Histogram of the FAI total scores. FAI, Frenchay Activities Index.

Histograms of the FAI total scores in the overall cohort, females, and males are depicted in Figure 1. Patients with scores between 24 and 27 were the most frequent scores in both females and males. In FAI, the components that were significantly commoner in females than males included preparing meals, washing up, washing clothes, light housework, heavy housework, and local shopping. Conversely, the components that were significantly more common in males than females included driving or bus travel, outings or car rides, household or car maintenance, and gainful work (Supplemental Table S1).

### 3.3. Cut-Off Values of FAI Score for All-Cause Mortality and Differences in Characteristics between the Groups

**Figure 2:**
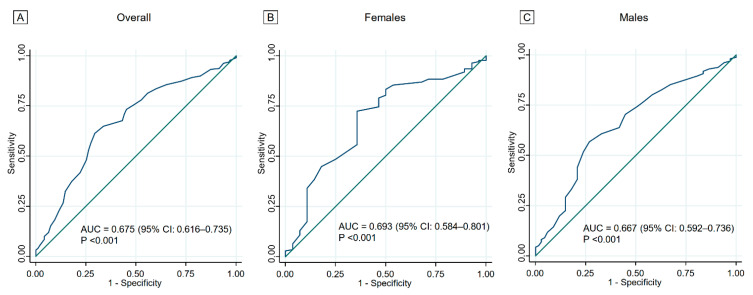
Receiver-operating characteristic curves of the FAI. The cut-off values of the FAI score for all-cause mortality were 23 points in the overall cohort (**A**), 22 points in females (**B**), and 23 points in males (**C**). AUC, area under the curve; FAI, Frenchay Activities Index.

The ROC curve of the FAI score for all-cause mortality is depicted in Figure 2. The AUCs were 0.675 (95% confidence interval [CI]: 0.616–0.735; *p* < 0.001) in all patients, 0.693 (95% CI: 0.584–0.801; *p* < 0.001) in females, and 0.664 (0.592–0.736; *p* < 0.001) in males. The cut-off values of the FAI score for all-cause mortality based on the Youden index were 23 points in the overall cohort, 22 points in females, and 23 points in males. We defined scores below the cut-off points for the respective sex as low FAI scores; consequently, 216 (44.1%) patients were included in the low FAI score group.

Compared to the patients in the high FAI score group, those in the low FAI score group were older; more likely to be female; had lower BMI; had more symptomatic and higher percentages of hypertension, diabetes mellitus, and ischemia; lower serum levels of albumin, creatinine, eGFR, BNP; and higher AHEAD score (Table 1). No significant differences in LVEF and medications were identified.

### 3.4. Association between FAI Score and Outcomes

**Figure 3:**
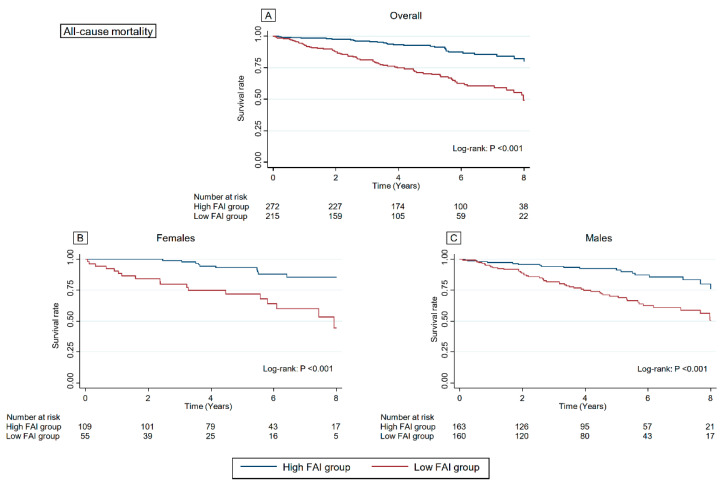
Kaplan–Meier curve for all-cause mortality according to the FAI in the overall cohort, females, and males. The all-cause mortality was significantly higher in the low FAI score group than those in the high FAI score group in the overall cohort (**A**), females (**B**), and males (**C**). FAI, Frenchay Activities Index.

**Figure 4:**
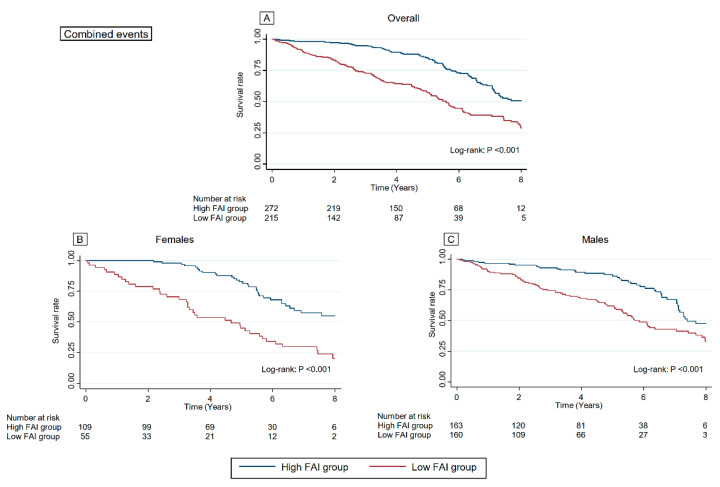
Kaplan–Meier curve for combined events according to the FAI in the overall cohort, females, and males. The rate of combined events was significantly higher in the low FAI score group than those in the high FAI score group in the overall cohort (**A**), females (**B**), and males (**C**). FAI, Frenchay Activities Index.

A total of 95 (19.4%) deaths due to all causes and 213 (43.5%) combined events (all-cause death and readmissions due to HF) were noted over a median follow-up period of 4.8 years (IQR: 2.3–6.6 years). Kaplan–Meier survival curve and log-rank test are summarised in Figure 3 and Figure 4. The all-cause mortality and rate of combined events were significantly higher in the low FAI score group than those in the high FAI score group in the overall cohort, females, and males (All *p* < 0.001, respectively). Table 2 summarises the results of Cox regression analysis for all-cause mortality and combined events. Even after adjusting for confounders including the AHEAD score, FAI was a significant and independent predictor of all-cause mortality and combined events (*p* = 0.002 and <0.001, respectively) in our cohort. Sensitivity analysis did not reveal any noticeable effect on the estimated association between FAI score and all-cause mortality (adjusted HR: 0.957; 95% CI: 0.933–0.982; *p* = 0.001).

**Figure 5:**
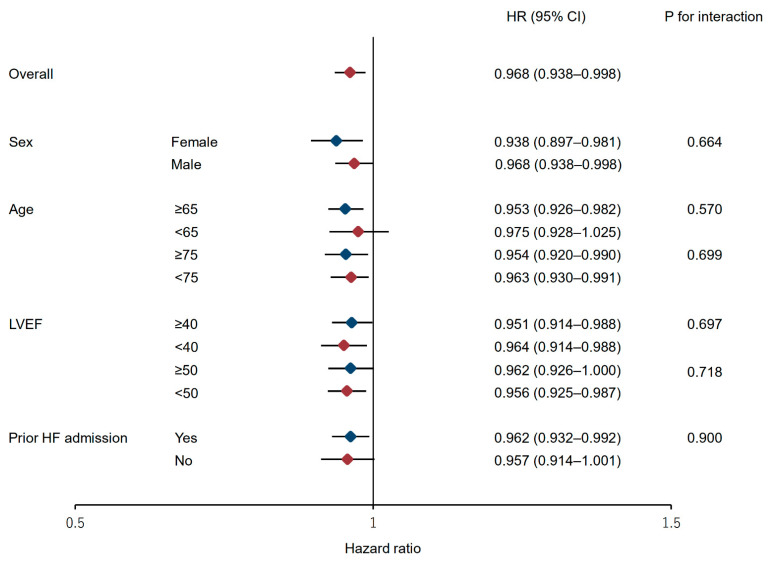
Forest plots of subgroup analyses of the associations between the FAI and all-cause mortality. All subgroups were adjusted for age, sex, body mass index, New York Heart Association functional classification Ⅲ/Ⅳ, LVEF, prior HF readmission, log-transferred B-type natriuretic peptide, β-blockers, and AHEAD score. CI, confidence interval; HF, heart failure; HR, hazard ratio; LVEF, left ventricular ejection fraction.

Low FAI score was consistently associated with high all-cause mortality across various subgroups, even after adjusting for confounders (Figure 5).

## 4. Discussion

The present study is the first report to investigate the association between IADL after outpatient cardiac rehabilitation and outcomes in patients with HF. The primary findings of our results are as follows: (1) the cut-off values of FAI for all-cause mortality were similar between the sexes (22 points in females and 23 points in males), although the FAI scores were relatively higher in females than those in males; (2) FAI predicted all-cause mortality and combined events; and (3) FAI also demonstrated a favourable prognostic capability in various subgroups in HF. These results suggest that IADL assessed using FAI constitute a favourable prognostic marker in patients with HF who completed outpatient cardiac rehabilitation and a useful outcome variable that should be assessed in clinical practice.

Several previous studies have investigated the importance of IADL in patients with cardiac disease. Lo et al. used the Cardiovascular Health Study (CHS) database, which is a prospective population-based observational study in the US, and reported that IADL impairment was associated with all-cause mortality in participants aged 65 years or older with incident HF [11]. Additionally, using the same database, Bowling et al. demonstrated that IADL predicted HF and were associated with all-cause mortality in community-dwelling individuals aged 65 years and older who had not been diagnosed with HF [9]. However, the median age of patients in these reports was 74 years; however, the reports did not include an age group that is assumed to be likely to perform IADL at a high frequency, and no analysis between the sexes was performed. Additionally, rehospitalization is an important outcome in HF [19,20,21,22]; however, the association between IADL and combined events, including rehospitalization, has not been investigated. Furthermore, we demonstrated the association between IADL and outcomes after completion of outpatient cardiac rehabilitation in patients hospitalized for HF. This is the first report to provide evidence regarding the importance of IADL as an outcome of outpatient cardiac rehabilitation.

Although the association between IADL and outcomes has been reported previously in community-dwelling individuals and individuals with some comorbidities [9,10,11,12,23,24,25,26], the underlying mechanisms remain unclear. Nevertheless, we evaluated IADL using the FAI, which is characterised by scoring each IADL item on frequency rather than simple presence or absence. This may be related to the quantity of physical activity, which is known to be associated with the prognosis in patients with HF through a variety of mechanisms, including vascular endothelial function [27] and oxidative stress [28]. In the present study, we evaluated FAI at the end of outpatient cardiac rehabilitation when most patients are considered to be stable, and the outcomes may have been poor because of low physical activity despite stable HF. Additionally, it is reported that physical function [29], cognitive function [30], and social status [31] are associated with IADL. Matsue et al. reported that overlapping physical, cognitive, and social frailty in patients with HF was associated with a poor prognosis [32,33], which may be another reason why poor IADL is associated with a poor outcome. Furthermore, females were found to perform items related to household work more frequently, while males performed items that were relatively overloaded more frequently. Since there was no difference in the prognostic capability between the sexes, a decrease in the frequency of items that do not differ between sexes could be associated with a poorer outcome.

We also calculated the cut-off values for FAI for each sex as well as the overall cohort. Very few reports have addressed the cut-off values for FAI; while only a few reports have assessed patients with stroke [34], none of them have suggested cut-off values for outcomes in patients with HF. The cut-off values in the present study may provide a reference value for risk stratification upon completion of outpatient cardiac rehabilitation. However, the cut-off values were calculated based on a small sample size at a single centre and are insufficiently validated; further validation studies and identification of subgroup cut-off values are warranted.

The strength of this study was the identification of the association between IADL assessed using the FAI score and all-cause mortality and combined events at the end of cardiac rehabilitation as well as the estimation of cut-off values of the FAI score. Furthermore, the association between IADL and outcomes and the sex-specific cut-off scores are important for risk stratification. Another strength of the study is that although it was a retrospective observational study, it included a long-term follow-up.

However, this study has several limitations. First, this was a single-centre retrospective study with a small sample size. The validity of the present results in other countries and populations requires further investigations. In addition, the dataset used in this study is rather old, and few internal medications that improve prognosis including angiotensin receptor neprilysin inhibitor and sodium-glucose cotransporter 2 inhibitors, which are currently the mainstay of heart failure medications [35,36,37], were used. This may affect the results. Therefore, multicentre prospective observational studies should be conducted. Second, this study examined IADL at the end of outpatient cardiac rehabilitation; however, there are no data regarding individual differences in the frequency and content of outpatient rehabilitation. Additionally, the frequency of outpatient cardiac rehabilitation visits in Japan has been reported to be low [38], and the results of this study may represent a population with relatively favourable compliance with the rehabilitation programme. Third, this study evaluated the IADL at only one point, and improvement in IADL does not indicate a better outcome. Therefore, it is necessary to examine the relationship between changes in IADL between two time-points—before hospitalization and/or early discharge—and outcomes. Finally, this study does not have data on the frequency and actual implementation of outpatient rehabilitation. These may have impact on IADL frequency and outcomes, and are an issue for future study.

## 5. Conclusions

Higher IADL frequency assessed using the FAI score at the end of cardiac rehabilitation is associated with a favourable outcome in patients with HF, irrespective of sex. The IADL level is generally higher in females than that in males, but appears to be a useful marker for risk stratification at the end of cardiac rehabilitation in both females and males.

## Figures and Tables

**Table 1:** Patient characteristics.

	Missing Data	Overall (N = 490)	High FAI Score (N = 274)	Low FAI Score (N = 216)	*p* Value
Age, years	0	69 (58–76)	67 (54–74)	72 (63–78)	<0.001
Female, n (%)	0	166 (33.9)	110 (40.1)	56 (25.9)	0.001
BMI, kg/m^2^	0	21.9 (19.5–24.2)	22.3 (19.7–25.3)	21.4 (19.3–23.7)	0.007
Etiology of HF, n (%)	0				
Ischemic		175 (35.7)	94 (34.3)	81 (37.5)	0.102
Hypertension		122 (24.9)	76 (27.8)	46 (21.3)	
Valvular		69 (14.1)	41 (15.0)	28 (13.0)	
Others		124 (25.3)	63 (23.0)	61 (28.2)	
NYHA functional class, n (%)	0				<0.001
Ⅰ/Ⅱ		345 (490)	225 (82.1)	120 (55.6)	
Ⅲ/Ⅳ		145 (29.6)	49 (17.9)	96 (44.4)	
LVEF, %	27	48 (33–59)	50 (35–61)	47 (31–58)	0.129
LVEF <40, n (%)		173 (35.3)	90 (32.8)	83 (38.4)	0.293
LVEF ≥50, n (%)		223 (45.5)	129 (47.1)	94 (43.5)	0.366
Comorbidities, n (%)					
Hypertension	0	308 (62.9)	159 (58.0)	149 (69.0)	0.013
Diabetes mellitus	0	171 (34.9)	78 (28.5)	93 (43.0)	0.001
Dyslipidemia	0	240 (49.0)	128 (46.7)	112 (51.9)	0.259
Atrial fibrillation	0	68 (13.9)	38 (13.9)	30 (13.9)	0.992
Ischemic etiology	0	203 (41.4)	100 (36.5)	103 (47.7)	0.013
Prior HF admission, n (%)	0	243 (50.0)	126 (46.0)	117 (54.2)	0.072
Current smoker, n (%)	0	87 (17.8)	45 (16.4)	42 (19.4)	0.336
Laboratory data					
Hemoglobin, g/dL	1	12.3 (10.8–14.0)	12.6 (11.1–14.0)	12.1 (10.4–14.1)	0.068
Albumin, g/dL	1	3.8 (3.4–4.0)	3.8 (3.4–4.1)	3.7 (3.4–4.0)	0.036
Creatinine, mg/dL	1	0.98 (0.80–1.34)	0.92 (0.77–1.24)	1.07 (0.86–1.55)	<0.001
eGFR, mL/min/1.73 m^2^	1	54.6 (37.7–69.6)	57.7 (43.3–72.6)	50.3 (28.1–65.6)	<0.001
Sodium, mEq/mL	1	139 (136–140)	139 (136–141)	138 (136–140)	0.184
BNP, pg/mL	34	219 (104–463)	191 (94–369)	278 (126–684)	<0.001
Medications, n (%)					
ACEI/ARB	0	418 (85.3)	237 (86.5)	181 (66.1)	0.402
β blocker	0	376 (76.7)	218 (79.6)	158 (73.1)	0.095
MRA	0	220 (44.9)	118 (43.1)	102 (47.2)	0.358
Diuretics	0	409 (83.5)	224 (81.8)	185 (85.6)	0.100
AHEAD score	0	2 (1–3)	1 (1–2)	2 (1–3)	<0.001
FAI	0	24 (16–29)	29 (26–32)	15 (10–19)	<0.001

ACEI, angiotensin-converting enzyme inhibitor; ARB, Angiotensin II Receptor Blocker; BMI, body mass index; BNP, B-type natriuretic peptide; eGFR, estimated glomerular filtration rate; FAI, Frenchay Activities Index; HF, heart failure; LVEF, left ventricular ejection fraction; MRA, mineralocorticoid receptor antagonist; NYHA, New York Heart Association.

**Table 2:** Cox proportional hazard models of the FAI for all-cause mortality and combined events.

Outcomes	No. of Events/Cases	Event Rate per 100 Persons-Years	HR (95% CI)	*p* Value
All-cause mortality (Per 1 point increase)	95/490 (19.4%)	4.2	0.961 (0.937–0.986)	0.002
High FAI score	28/274 (10.2%)	1.0	1 (Reference)	
Low FAI score	67/216 (31.0%)	6.5	2.69 (1.64–4.44)	<0.001
Combined events (Per 1 point increase)	213/490 (43.5%)	9.1	0.968 (0.952–0.985)	<0.001
High FAI score	89/274 (32.5%)	6.8	1 (Reference)	
Low FAI score	114/216 (52.8%)	11.0	1.552 (1.054–2.353)	0.033

Adjusted for age, sex, body mass index, left ventricular ejection fraction, brain-natriuretic peptide, New York Heart Association functional classification, prior heart failure admission, β blocker, and AHEAD score. High FAI score was defined as above the cut-off value for each sex, and low FAI score was defined as that below the cut-off value. CI, confidence interval; FAI, Frenchay Activities Index; HR, hazard ratio.

## Data Availability

The data used in this article cannot be shared publicly in view of the privacy of individuals that participated in the study. The data will be shared upon reasonable request to the corresponding author.

## Supplementary Materials

The following supporting information can be downloaded at: https://www.mdpi.com/article/10.3390/jcdd9090289/s1, Table S1: The FAI component values in females and males.
