# Supplementary material for: Sex Differences in Frequency of Instrumental Activities of Daily Living after Cardiac Rehabilitation and Its Impact on Outcomes in Patients with Heart Failure
Source: J Cardiovasc Dev Dis. 2022 Aug 31;9(9):289. doi: 10.3390/jcdd9090289 (PMC9502306; doi:10.3390/jcdd9090289)
Supplement: Supplementary file 1 [file jcdd-09-00289-s001.zip › jcdd-1809421-supplementary.pdf]

**Supplementary Materials:**

Table S1. The FAI component values in females and males.

| Component of the FAI | Female | Male | P value |
|---|---|---|---|
| 1. Preparing meals | 3 (2–3) | 1 (0–3) | <0.001 |
| 2. Washing up | 3 (3–3) | 2 (0–3) | <0.001 |
| 3. Washing clothes | 3 (2–3) | 0 (0–3) | <0.001 |
| 4. Light house work | 3 (2–3) | 2 (0–3) | <0.001 |
| 5. Heavy house work | 2 (0–3) | 1 (0–3) | 0.025 |
| 6. Local shopping | 3 (2–3) | 3 (1–3) | 0.008 |
| 7. Social outings | 2 (0–3) | 2 (1–3) | 0.523 |
| 8. Walking outside for >15 min | 3 (2–3) | 3 (2–3) | 0.152 |
| 9. Pursuing hobby | 1 (0–3) | 1 (0–3) | 0.941 |
| 10. Driving or bus travel | 3 (1–3) | 3 (2–3) | <0.001 |
| 11. Outings or car rides | 0 (0–0) | 0 (0–1) | 0.003 |
| 12. Gardening | 0 (0–1) | 0 (0–1) | 0.735 |
| 13. Household or car maintenance | 0 (0–1) | 1 (0–2) | <0.001 |
| 14. Reading books | 2 (0–3) | 2 (0–3) | 0.621 |
| 15. Gainful work | 0 (0–0) | 0 (0–3) | <0.001 |

Values are expressed as median (interquartile range).

FAI, Frenchay Activities Index.
